# Symptom-reducing actions: a concept analysis in the context of chronic obstructive pulmonary disease

**DOI:** 10.1080/17482631.2017.1387452

**Published:** 2017-10-15

**Authors:** Ann-Britt Zakrisson

**Affiliations:** ^a^ University Healthcare Research Centre, Faculty of Health and Medicine, Örebro University, Örebro, Sweden

**Keywords:** Chronic obstructive pulmonary disease, concept analysis, nursing, person-centred care, self-care, self-management

## Abstract

Patients with Chronic Obstructive Pulmonary Disease (COPD) have multiple symptoms. Nursing care is based on six core competencies and one of them is person-centred care that includes the aspect of professional symptom relief. The aim was to clarify a meaning of the concept of Symptom-reducing actions in the context of COPD. Databases MEDLINE and CINAHL were searched between 1982 and February 2016 and 26 publications were found. Two dictionaries and three books were investigated. The method of Walker & Avant was followed. The use of the concept of Symptom-reducing actions may be categorized by the sub-concepts of supervision, information, and patient education. Exploration of defining attributes was symptom management, instructions, support, motivation, explanation, advice, teaching, and learning. Antecedent occurrences were related to factors that affect the patient’s level of function such as physical performance and cognitive function. Symptom-reducing actions offer a way to support patients with COPD in self-management. Symptom-reducing actions can mediate facts in a purposeful process performed by the nurse to enable the patient to take control over and manage unpleasant symptoms by a person-centred, planned process. The nurse can achieve this via supervision, information, and patient education with an integrated emotional component. Evaluating the outcomes is needed.

## Introduction

Patient education and self-management support is an important part of nursing care. To patients with Chronic Obstructive Pulmonary Disease (COPD) the education provided by the nurses is important in strengthening their ability for self-care (Orem, Renpenning, & Taylor, 1995). One large part of COPD treatment is exercise, aimed to reduce symptoms and improve quality of life and capacity in daily life (GOLD, 2015). Nurses are responsible to optimize patients with COPD functional status by educating and encouraging participation in physical activities. When performing physical activity patients have to struggle with symptoms like breathlessness, cough, lack of energy, and so on (Theander et al., 2014). Nursing care is, among other things, based on six core competencies (Leksell & Lepp, 2013; QSEN, 2007): person-centred care (is based from the patient’s story), teamwork collaboration (to recognize gaps in the team and initiate inter-professional teamwork), evidence-based practice (to contribute in developing new guidelines and participate in research), quality improvement (to initiate development of quality in work), safety (to be responsible and lead systematic work in patient safety), and informatics (to support and guide the patients to sort digital information and participate in developing eHealth) which include satisfaction with care, involvement in care, feeling of well-being, and creating a therapeutic environment described as one in which decision-making is shared, staff relationships are collaborative, leadership is transformational, and innovative practices are supported (Leksell & Lepp, 2013; QSEN, 2007). Person-centred care includes the aspect of professional symptom relief (McCormack & McCance, 2006) which is important in this case when educating patients with COPD, both to use person-centred care and symptom relief. COPD cannot be cured, but is preventable and treatable (GOLD, 2015). It is essential that nurses caring for persons with COPD provide patient education in self-management including relief symptoms. Patient education should not only mediate facts and information, but also include an emotional component to address the patients’ feelings and reactions (Klang Söderkvist, 2008; Lejsgaard Christensen, Huus Jensen, & Karman, 2004; Rankin, Duffy Stallings, & London, 2005). The work of COPD nurses includes continuous education, information provision and supervision. Patient education is a substantial part of this work, and in many cases is primarily a question of self-care (Orem et al., 1995).

Patients with COPD have multiple symptoms, the most frequent being shortness of breath, cough, lack of energy, dry mouth, numbness or tingling in the hands and feet, and pain (Theander et al., 2014). Pulmonary rehabilitation is known to be one of the most effective treatments of COPD, and is defined by American Thoracic Society/European Respiratory Society (ATS/ERS) as being ‘designed to reduce symptoms, optimize functional status, increase participation and reduce health care costs through stabilizing or reversing systemic manifestations of the disease’ (ATS/ERS, 2006, p. 1391). The concept of patient education is one of many in this field, and the concept of *Symptom-reducing actions* is a suitable way to describe the specific work of COPD nurses. Reduction of symptoms is desirable for both patient and nurse and it is important to investigate what the nurse’s responsibility is and what his or her role is in self-management support. The aim of this article is to clarify a meaning of the concept of Symptom-reducing actions in the context of COPD.

## Method

**Figure 1.**
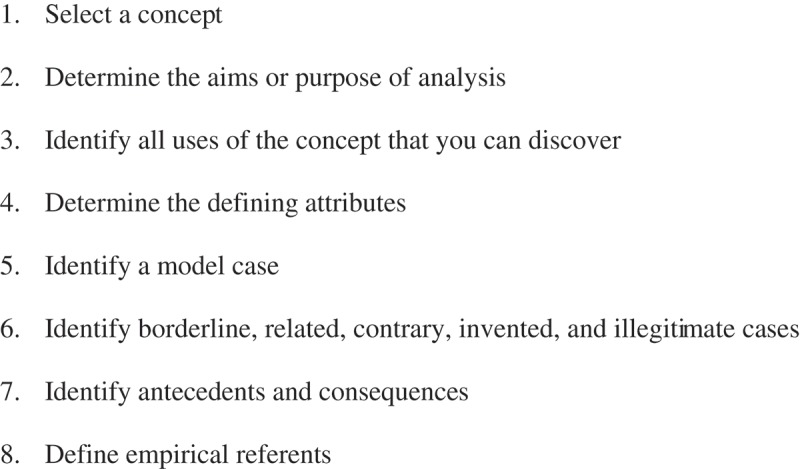
Walker & Avant’s approach to concept analysis by eight steps.

Walker & Avant’s (2011) approach to concept analysis was used as a method to define the concept of Symptom-reducing actions. Eight steps are used in the process (Figure 1). In brief the method is sufficient to start thinking carefully about language and its uses in health care. Concept analysis can clarify words or terms used in communication as to mean the same thing. It renders theoretical as well as operational definitions for use in theory and research. One advantage is that the analysis can help clarify those terms in nursing that have become slang and jargon and therefore have missed their meanings. Examining the structure and function of a concept is the aim of the analysis.

### Material

Two databases (MEDLINE and CINAHL) that revealed 26 publications (Table I), 2 dictionaries (Encarta World English Dictionary, 2017; Wordsmyth English Dictionary), and 3 books on how to provide patient education (Klang Söderkvist, 2008; Lejsgaard Christensen et al., 2004; Rankin et al., 2005) were investigated to clarify the concept of Symptom-reducing actions. The databases were searched for articles written in English and published between 1982 and February 2016, using the search terms *concept analysis, Symptom-reducing actions, symptom, patient education, information, supervise, rehabilitation,* and *nursing*, both separately and in combination. When adding the term *COPD* there were no results (Table II). The selection took place in three steps: reading title, then abstract, then reading relevant studies in full text. The reference lists of the articles retrieved were then searched for related studies. Nothing was found in searching for the concept of Symptom-reducing actions itself, but the analysis included all the articles which were particularly related to Symptom-reducing actions, for example those discussing the meaning of symptoms. Articles were excluded if they only used the concepts of symptoms, patient education, information, and supervision in an implicit way; if they only used symptoms as outcomes in intervention studies; or if they only covered patient education, information, and supervision in relation to nursing students. This approach produced a large number of articles and only those relating to the concept in reviews, middle-ranged theories, and concept analysis were selected.

**Table I.** Description of the included 26 articles.

Author Year Journal	Design/objective	Findings	Conclusion
Armstrong, 2003*Oncol Nurs Forum*	Concept analysis of symptom experience	Various individual factors interact to produce symptoms in individuals, and the occurrence of symptoms can influence functional health status.	Symptoms experience is the perception of the frequency, intensity, distress, and meaning of symptoms as they are produced and expressed. Symptoms are multiplicative in nature and may act as catalysts for the occurrence of other symptoms. Antecedents to the symptoms experience include demographic, disease, and individual factors. Consequences include the impact on mood state, psychological status, functional status, quality of life, disease progression, and survival.
American Thoracic Society/European Respiratory Society (ATS/ERS) 2006 *Am J Respir Crit Care Med*	Statement of pulmonary rehabilitation (description of symptoms, outcomes, measurement)	Provides a definition of Pulmonary rehabilitation. Patients with COPD often suffer from exercise intolerance, dyspnea, fatigue, anxiety, depression, poor motivation, cardiac ischemia, musculoskeletal problems, osteoporosis, nutritional problems, being underweight or overweight, urine incontinence, decreased quality of life, sexual problems, concentration/memory/cognitive dysfunction, social isolation, feelings of guilt, and sleep disorders. Outcomes that are important, such as control of symptoms, the ability to perform daily activities, exercise performance, and improved quality of life. Several COPD-specific instruments are available to measure the effectiveness of symptom-reducing actions.	The evidence for improvement in exercise endurance, dyspnoea, functional capacity, and quality of life is stronger for rehabilitation than for almost any other therapy in COPD, and documentation of its favourable effect on health care utilization is increasing. The success of pulmonary rehabilitation stems from its favourable inﬂuence on systemic effects and comorbidities associated with chronic lung disease.
Dodd et al., 2001 *J Adv Nurs*	Concept model of symptom management	The goal of symptom management is to avert or delay a negative outcome through biomedical, professional, and self-care strategies. Management begins with assessment of the symptom experience from the individual’s perspective. Assessment is followed by identifying the focus for interven tion strategies.	The symptom management model continues to evolve as a framework for understanding symptoms, designing, and testing management strategies and for evaluating outcomes.
Erdley, 2005 *Comput Inform Nurs*	Concept development of nursing information	Information is the function of the communication process between interacting persons, whether verbal or written. Time has an impact on its value. Communication, context, form, and value describe information. Nursing information is unique to each individual, and so a variety of forms will exist. Through communication, nurses become aware of their ability to provide support, heighten awareness, and provide anticipatory guidance to those in their care. Nursing information emerges as a multidimensional concept influenced by context and by individual perceptions and understanding.	Nursing information emerged as a multidimensional concept influenced by context and individual perceptions and understanding. Technologies like computers and computer networks are tools used by nurses and other healthcare practitioners that assist with patient care and information management.
Franek, 2013 *Ont Health Techno Assess Ser*	Systematic review of self-management support	Support in self-management is commonly used as a structured way of helping patients learn to better manage their chronic disease.	The Stanford Chronic Disease Self-Management Program (CDSMP) led to short-term improvements across a number of health status measures (including some measures of health-related quality of life), healthy behaviours, and self-efficacy compared to usual care. There was no evidence to suggest that the CDSMP improved health care utilization.
Fu et al., 2004 *Oncol Nurs Forum*	Concept analysis of symptom management	Symptom management is a dynamic and multidimensional process in which patients intentionally and purposefully act on and interact with the perception (or previous perception) of the symptoms in order to initiate activities or direct others to perform activities to relieve or decrease distress from a symptom, and to prevent its occurrence.	The essential attributes of symptom management are subjectivity, experientiality, intentionality, multidimensionality, dynamic process, and positive and negative outcomes.
Haworth & Dluhy, 2001 *J Adv Nurs*	Concept model of holistic symptom management	The patient and the nurse require varying amounts of time to allow for the revelation, initial understanding, clarification, and interpretation of the symptom experience. The relationship between nurse and patient takes time to form, and is shaped by the degree of mutuality and trust. Both parties must have respect and accountability. Effective symptom management is dependent on the nurse hearing and attending to the lifeworld of the client.	Attending to key areas of influence in the interaction process facilitates the achievement of desired outcomes in symptom management: accurate diagnosis, symptom relief, and agreement on a course of action. The dominance of chronic illness in health care, and the centrality of the symptom experience underscores the value of sensitizing nurses to these issues.
Henly et al., 2003 *Nurs Res*	The concept of time in symptom experiences.	Symptom experiences in time (SET) theory conceives the symptom experience as a flow process that explicitly incorporates temporal dimensions. Four dimensions of time are recognized: clock-calendar; biologic-social, perceived; and transcendent time. The four temporal dimensions are placed against a backdrop of ‘meaning-in-time’ that brings forth the potential for transformation in a symptom experience. Increasing sophistication in design, measurement, and data analysis is required to test and evaluate SET theory-based propositions. Symptom management involves the transformation of unpleasant sensations to action or non-action via decisions about seriousness, unpleasantness, explicability, and treatability.	The SET theory extends previous work by incorporating multiple temporal dimensions that reflect the human experience of health and illness manifested in the expression and management of symptoms.
Hutchinson & Wilson, 1998 *Sch Inq Nurs Pract*	The Theory of Unpleasant Symptoms	The caregiver and the social and environmental context, called situational factors are of importance in the theory.	When symptoms are examined in their entirety, and nursing interventions take the interactive nature of symptoms, influencing factors and symptom consequences/performance outcomes into consideration, interventions should be client specific and, therefore, more effective.
Jolly et al., 2016 *Int J Chron Obstruct Pulmon Dis*	Systematic review and meta-analysis of Self-management of health care behaviours	Self-management interventions had a minimal effect on hospital admission rates. Multicomponent interventions improved. HRQoL Exercise was an effective individual component. Self-management interventions involve collaboration between healthcare professional and patient, so the patient acquires and demonstrates the knowledge and skills required to manage their medical regimens, change their health behaviour, improve control over their disease, and improve their condition.	While many self-management interventions increased HRQoL, little effect was seen on hospital admissions.
Jonkman et al., 2016 *Patient Educ Couns*	Systematic review and meta-regression analysis of components of self-management interventions that improve HRQoL	A self-management intervention includes several components: stimulation of symptom monitoring, education in problem solving skills regarding acute exacerbations or symptoms, resource utilization, enhancement of medication adherence, physical activity, dietary intake, and smoking cessation. Self-management interventions showed great diversity in mode, content, intensity, and duration. Although self-management interventions overall improved HRQoL at 6 and 12 months, meta-regression showed counterintuitive negative effects of standardized training of interventionists and peer interaction on HRQoL at 6 months.	Self-management interventions improve HRQoL at 6 and 12 months, but interventions evaluated are highly heterogeneous. No components were identified that favourably affected HRQoL. Standardized training and peer interaction negatively influenced HRQoL, but the underlying mechanism remains unclear.
Kim et al., 2005 *Cancer Nurs*	Concept analysis of symptom clusters	A symptom cluster is defined as consisting of two or more symptoms that are related to each other and that occur together. Symptom clusters are composed of stable groups of symptoms, are relatively independent of other clusters, and may reveal specific underlying dimensions of symptoms. Relationships among symptoms within a cluster should be stronger than relationships among symptoms across different clusters. Symptoms in a cluster may or may not share the same aetiology. Symptom should be broadened to include both subjective (self-reported) symptoms and objective (observed) signs.	Team members promote self-management and have strategies to reduce even those symptoms that occur together (i.e., symptom clusters).
Larson, 1994 *Image J Nurs Sch*	A model for symptom management	Symptom management is a challenging experience for patients, families, and health care professionals. The model, focus on symptom experience, management strategies, and outcomes.	The model is applicable to practice and research, and is currently being used in a variety of studies and settings.
Lenz et al., 1995 *ANS Adv Nurs Sci*	Theory development of unpleasant symptoms	Unpleasant symptoms are illness-related rather than treatment-related, and there may be relationships between them. Physiological, psychological, and situational factors affect the patient’s performance, including functional status, cognitive functions, and physical performance.	A sustained substantive example is provided by the beginning development of a theory of unpleasant symptoms.
Lenz et al., 1997 *ANS Adv Nurs Sci*	The middle-range theory of unpleasant symptoms	Symptoms can occur alone or in isolation from one another, but if multiple symptoms occur simultaneously they are likely to catalyse each other and further worsen the overall experience. Several dimensions are common across symptoms and clinical populations: intensity (strength and severity); timing (duration and frequency of occurrence); level of distress perceived (degree of discomfort or bothersomeness); and quality. People differ in their ability to discern symptoms.	Revisions have resulted in a more accurate representation of the complexity and interactive nature of the symptom experience. Examples are provided to demonstrate the implications of the revised theory for measurement and research, and its application in practice.
Parker et al., 2005 *J Nurs Scholarsh*	Review of Symptom interactions	The team members use observable action to recognize, alleviate, or eliminate symptoms. The nurse promotes self-management and has strategies to reduce symptoms.	These results indicate the need for further work and theory development in this area. The symptom interactional framework is a beginning conceptual perspective designed to facilitate this work. Implications for interdisciplinary translational research designed to optimize symptom management are discussed.
Piredda, 2004 *International Nursing Perspectives*	Concept analysis of patient education	Communication and dialogue are needed in patient education. The process is patient-centered, and needs interaction, time, partnership, and the patient’s readiness and willingness.	Patient education is a planned process of activities designed to enable people to improve knowledge, to acquire skills, and to facilitate voluntary adaptation of behaviours in order to restore, maintain, and improve health.
Posey, 2006 *Nurs Forum*	Concept exploration of symptom perception	Important aspects include the belief that an individual has about what a particular symptom means (cognitively and emotionally), the appraisal of the symptom based on past and present knowledge and experience, and the response or action of the individual based upon that meaning and appraisal. The individual is an active participant while experiencing the symptom.	Symptom perception and the related concepts have overlapping ideas but vary in meaning and in measurement. This concept analysis explored symptom perception and the related concepts and looked at each one critically in its applicability to a final working definition of symptom perception.
Powell & Gibson, 2007 *The Cochrane Library*	Systematic review of self-management education	The nurse clarifies the meaning of something and recommends actions with advice. Patient education is aimed at helping patients gain the motivation, skills, and confidence to control their disease, and gain both a written and a verbal form.	Optimal self-management allowing for optimization of disease control may be conducted by either self-adjustment with the aid of a written action plan or by regular medical review. Individualized written action plans based on symptoms. Reducing the intensity of self-management education or level of clinical review may reduce its effectiveness.
Reach, 2009. *Patient Educ Couns*	Trans-theoretical analysis of obstacles to patient education	Considering future patients’ adherence to educational programmes and long-term therapies, it must be focusing on patients’ projects.	The very concept of prevention entails features that jeopardize the efficiency of educational programmes used for its implementation. In chronic diseases, designing programmes proposing concrete and short-term preventive measures may represent a way to overcome this obstacle. Habit may be used to reinforce connectedness which forms personal identity. Thus, taking into account this temporal dimension of educational programmes is essential.
Roman-Rodriguez et al., 2016 *NPJ Prim Care Respir Med*	RCT for assessment tools in symptoms	Several COPD-specific instruments are available to measure the effectiveness of educational activities such as COPD Assessment Test (CAT) and modified Medical Research Council dyspnoea scale (mMRC).	An educational intervention programme targeted on primary care physicians enhances the use of respiratory health status tools and promotes behavioural changes.
Stamler, 1996 *J Holist Nurs*	Concept analysis of enablement in patient education.	Patient education is one of the interventions that are formally and informally directed at enablement, and the nurse plays the role of enabler. The process is patient-centred, and needs interaction, time, and partnership.	An analysis that resulted in a definition of enablement and the identification of three components: means; abilities; and opportunities.
Teel et al., 1997 *Image J Nurs Sch*	Theory of symptom interpretation.	Symptom interpretation model is based on an illness representation model, knowledge structures theory, and propositions about reasoning. Individuals name and assign meaning to environmental stimuli. Based on this interpretation, behaviours are selected for symptom management.	Symptom familiarity reinforces patterns about symptom management. Symptom interpretation model enriches understanding of symptom experiences. The intra-individual perspective, is essential to successful symptom management.
Timmins, 2006 *Int J Nurs Pract*	Concept exploration of information need.	Information seeking is the most frequent method used to cope with a stressful event about which information is limited, and in this case communication requires professional action.	Information need emerged as a want or desire for information to be shared by professionals using appropriate communication skills. Information-seeking behaviour manifests in individuals as a response to a stimulus that is perceived as either a challenge or a threat.
Tveiten, 2005 *J Nurs Manag*	Concept evaluation of supervision.	Supervision can be defined as a formal, pedagogical, relational enabling process, related to professional competence. Relationship and dialogue are central aspects. Supervision is based on theory and humanistic values, has a normative, formative and restorative function. The supervisor’s competence is of great importance. Supervision has unclear boundaries with concepts such as psychotherapy, consultation, and counselling.	Supervision can be defined as a formal, pedagogical, relational enabling process with the purpose to strengthen resources, enhance assertiveness, and improve independence and coping.
Yoon et al., 2006 *Int J Nurs Pract*	Concept exploration of patient education.	Patient education is a planned learning activity, purposefully designed and systematically implemented. It occurs over a period of time, and enables the learner to properly understand the information provided. It is flexibly and individually implemented to fulfil the patient’s unique needs.	Successful patient education includes enhanced patient participation in health care decision-making, improved commitment to treatment, increased patient satisfaction, increased ability to cope with illness, improved quality of life in patients and their families, and decreased anxiety.

**Table II.** Overview of literature search in MEDLINE and CINAHL articles written in English and published between 1982 and February 2016.

Keyword	Number of hits	Included
Concept analysis	54,458	
AND symptom AND nursing	111	14
AND patient education AND nursing	129	8
AND Information AND nursing	406	2
AND supervise	8	1
AND rehabilitation AND nursing	446	1

## Findings

### Use of the concept

Patients with COPD often suffer from exercise intolerance, dyspnea, fatigue, anxiety, depression, poor motivation, cardiac ischemia, musculoskeletal problems, osteoporosis, nutritional problems, being underweight or overweight, urine incontinence, decreased quality of life, sexual problems, concentration/memory/cognitive dysfunction, social isolation, feelings of guilt, and sleep disorders (ATS/ERS, 2006). In the literature, the concept of symptoms is mostly used in an implicit way, as symptom control, symptom perception, symptom interactions, symptom relief, symptom consequences, symptom distress, symptom containment, and symptom reporting.

In an interview study about COPD nurses’ experience of providing patient education to patients with COPD (Zakrisson & Hägglund, 2010), the nurses used terms such as *information, advice, educate, teaching, learning, explain, support, supervise, motivate,* and *tell*; they avoided using the term *patient education* itself. They were also very concerned about creating contact with the patient, and the results showed that they met the patients’ feelings and reactions in a good way. The nurses in the study occasionally reflected on their education, mainly when they had a feeling of inadequate achievement. Meeting the patient, creating contact, and reflecting on the education are strongly integrated parts of patient education (Klang Söderkvist, 2008; Lejsgaard Christensen et al., 2004; Rankin et al., 2005).

### Dictionary and thesaurus definitions

The dictionaries (Encarta World English Dictionary, 2017; Wordsmyth English Dictionary) were first used to create a base by defining the terms *supervision, information,* and *patient education*. These three terms seemed to be most linked to the concept of Symptom-reducing actions, as showed in the result of the interview study (Zakrisson & Hagglund, 2010).

*Supervision* was defined as watching over an activity or task being carried out by somebody else to ensure that it is performed correctly and being in charge of a group of people engaged in an activity or task to keep order or ensure that they perform it correctly.

*Information* was defined as definite knowledge acquired or supplied about something or somebody, the collected facts and data about a specific subject, and the communication of facts and knowledge.

*Patient education* had no distinct definition and had to be defined in two parts. A patient was defined as somebody receiving medical treatment. Education was defined as the imparting and acquiring of knowledge through teaching and learning, especially at a school or similar institution, and the knowledge or abilities gained through being educated, trained, and instructed in a particular subject (e.g., health matters).

From these actions, supervision, information, and patient education, the term *symptom* was the most carrying symbol for the concept of Symptom-reducing actions that would be analysed, i.e., the symptoms are what can be reduced by these actions performed by the nurse.

#### Symptom

Symptom was defined in the dictionaries as an indication of illness felt by the patient, or an indication of a disease or other disorder, especially one experienced by the patient (e.g., pain, dizziness, or itching) as opposed to one observed by the doctor; or a sign or indication of the existence of something, especially something undesirable (Encarta World English Dictionary, 2017); or an abnormal phenomenon caused by and indicating a disease or disorder (Wordsmyth English Dictionary). Its synonyms were: indication, sign, warning sign, indicator (Encarta World English Dictionary, 2017) (thesaurus); or warning, indication (Wordsmyth English Dictionary).

#### Reducing

To *reduce* was defined as to decrease and become smaller in size, number, extent, degree, or intensity, or to make something smaller in this way (Encarta World English Dictionary, 2017) (dictionary); or to make something less in amount or size (Wordsmyth English Dictionary) (definition). Its synonyms were: diminish, modify, lessen, lower, moderate, and decrease (Wordsmyth English Dictionary). To *simplify something* was defined as to make something simpler, especially by extracting or summarizing essential components (Encarta World English Dictionary, 2017) (dictionary).

#### Actions

An *action* was defined as *doing something toward a goal*: the process of doing something in order to achieve a purpose (Encarta World English Dictionary, 2017) (dictionary); or as *something done*: something that somebody or something does (Encarta World English Dictionary, 2017) (dictionary); or as something happening or being done (Wordsmyth English Dictionary). Its synonyms were practice, labour, work, activity, act, deed, operation, and execution (Wordsmyth English Dictionary).

### Sub-concepts

**Figure 2.**
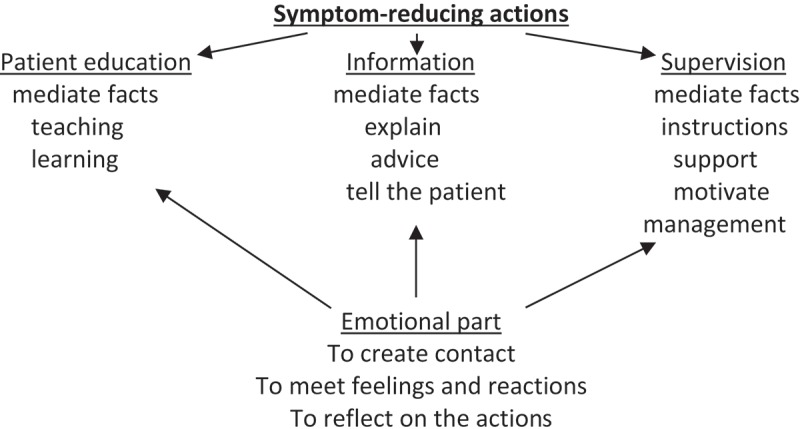
Symptom-reducing actions in relation to education, information, supervision, and the emotional part.

The three sub-concepts — *supervision, information*, and *patient education —*were used by the COPD nurses in the interview study when they described what they did and how they helped their patients with COPD regarding self-care (Zakrisson & Hagglund, 2010). The boundaries between these three sub-concepts are vague, and as a whole Symptom-reducing actions create an umbrella term for them. In the articles revealed by the database search and the three books, the *emotional component* (meet feelings and reactions, create contact and reflect on the patient education) was integrated in the authors’ analyses of their concepts (Figure 2).

***Supervision*** can be defined as an enabling process which is formal, pedagogical, and relational. It is an ongoing process providing help to understand something in order to increase self-awareness and coping. It is related to professional competence. The central aspects are relationship and dialogue; time, place, and duration are also important. Theory and humanistic values provide the basis for supervision. The supervisor has a normative (to guide), formative (to shape or form), and restorative (to strengthen) function. The competence of the supervisor is of great importance (Tveiten, 2005).

***Information*** is the function of the communication process between interacting persons, whether verbal or written (Erdley, 2005). Time has an impact on its value: there must be enough time to be able to listen and interact with the patient. Communication, context, form, and value describe information. Nursing information is unique to each individual, and so a variety of forms will exist. Through communication, nurses become aware of their ability to provide support, heighten awareness, and provide anticipatory guidance to those in their care (Erdley, 2005). Information seeking is the most frequent method used to cope with a stressful event about which information is limited, and in this case communication requires professional action (Timmins, 2006). Nursing information emerges as a multidimensional concept influenced by context and by individual perceptions and understanding (Erdley, 2005).

***Patient education*** is aimed at helping patients gain the motivation, skills, and confidence to control their disease, and again can take both written and verbal form (Powell & Gibson, 2007). This is one of the interventions that are formally and informally directed at enablement, and the nurse plays the role of enabler (Stamler, 1996). Communication and dialogue are needed (Piredda, 2004; Powell & Gibson, 2007; Stamler, 1996; Yoon, Conway, & McMillan, 2006). The process is patient-centred, and needs interaction, time, partnership (Piredda, 2004; Stamler, 1996), and the patient’s readiness and willingness (Piredda, 2004). Considering future patients’ adherence to educational programmes and long-term therapies, it is suggested that focusing on patients’ projects may be more relevant (Reach, 2009), which means that patient education must be more person-centred. ‘Patient education is a planned process of activities designed to enable people to improve knowledge, to acquire skills and to facilitate voluntary adaptation of behaviours in order to restore, maintain and improve health’ (Piredda, 2004, p. 69). Patient education is a planned learning activity, purposefully designed and systematically implemented. It occurs over a period of time, and enables the learner to understand properly the information provided. It is flexibly and individually implemented to fulfil the patient’s unique needs (Yoon et al., 2006).

### Defining attributes

Defining attributes were found for the three sub-concepts: *supervision*; *information*; and *patient education*. Below, some of the terms from the study of the COPD nurses are presented beneath the most appropriate sub-concept.

#### Supervision

The defining attributes that the COPD nurses mentioned of supervision were: *symptom management; instructions*; *support*; and *motivation*.

*Symptom management* involves the transformation of unpleasant sensations to action or non-action via decisions about seriousness, unpleasantness, explicability, and treatability (Henly, Kallas, Klatt, & Swenson, 2003). Support in self-management is commonly used as a structured way of helping patients learn to better manage their chronic disease (Franek, 2013). Self-management interventions involve collaboration between healthcare professional and patient, so the patient acquires and demonstrates the knowledge and skills required to manage their medical regimens, change their health behaviour, improve control over their disease, and improve their condition (Jolly et al., 2016). The team members use observable action to recognize, alleviate, or eliminate symptoms. They promote self-management and have strategies to reduce symptoms (Parker, Kimble, Dunbar, & Clark, 2005), even those symptoms that occur together (i.e., symptom clusters) (Kim et al., 2005). A self-management intervention includes several components: stimulation of symptom monitoring; education in problem-solving skills regarding acute exacerbations or symptoms; resource utilization; enhancement of medication adherence; physical activity; dietary intake; and smoking cessation (Jonkman, Schuurmans, Groenwold, Hoes, & Trappenburg, 2016). Situational factors such as caregiver characteristics and cultural background are important in reducing symptoms (Dodd et al., 2001; Hutchinson & Wilson, 1998). Assessment of symptoms should include the patient’s own assessment, clinical parameters, and observation by others (Teel, Meek, McNamara, & Watson, 1997). The goal is to avert or delay a negative outcome through biomedical, professional, and self-care strategies. This is a dynamic process which involves changing strategies over time or in response to a patient’s acceptance (or lack of acceptance) of the strategy (Dodd et al., 2001; Larson & UCSF, 1994). A more generic intervention that takes the approach of providing patients with education, self-care skills, and support is needed. It is important to include family members (Dodd et al., 2001). The patient and the nurse require varying amounts of time to allow for the revelation, initial understanding, clarification, and interpretation of the symptom experience (Haworth & Dluhy, 2001). Important aspects include the belief that an individual has about what a particular symptom means (cognitively and emotionally), the appraisal of the symptom based on past and present knowledge and experience, and the response or action of the individual based upon that meaning and appraisal. The individual is an active participant while experiencing the symptom (Posey, 2006). The relationship between nurse and patient takes time to form, and is shaped by the degree of mutuality and trust. Both parties must have respect and accountability. Effective symptom management is dependent on the nurse hearing and attending to the lifeworld of the client (Haworth & Dluhy, 2001). Symptom management is a dynamic and multidimensional process in which patients intentionally and purposefully act on and interact with the perception (or previous perception) of the symptoms in order to initiate activities or direct others to perform activities to relieve or decrease distress from a symptom, and to prevent its occurrence (Fu, LeMone, & McDaniel, 2004). Various individual factors interact to produce symptoms in individuals, and the occurrence of symptoms can influence functional health status (Armstrong, 2003). As a result, according to the analysis of earlier references, there seems to be a difference between symptom-management and Symptom-reducing actions. Symptom-management is the patient’s challenge and Symptom-reducing actions are the responsibility of the nurse, supporting the patients to manage their symptoms.

Nothing relevant to a concept analysis of *instruction* could be found in the database search; everything was related to nursing education and students. *Instruction* is a statement of command; a spoken or written statement of what must be done, especially delivered formally, with official authority, or as an order (Encarta World English Dictionary, 2017). The Wordsmyth dictionary defines it as directions or orders (Wordsmyth English Dictionary).

Nothing explicit regarding the concept of *motivation* was found in the database search; it was only used in an implicit way. *Motivation* is the act of giving somebody a reason to do something (Encarta World English Dictionary, 2017). Motivation is also a feeling of enthusiasm, interest, or commitment that makes somebody want to do something, or something that causes such a feeling (Wordsmyth English Dictionary).

Nothing relevant to a concept analysis of the attribute *support* could be found. *Support* is the encouragement given to someone during periods of stress or affliction (Wordsmyth English Dictionary). To support someone is to give them assistance or comfort when they are in difficulty or distress, and to give them active help, encouragement, or money (Encarta World English Dictionary, 2017).

#### Information

The defining attributes that the COPD nurses mentioned of information were *explanation* and *advice*.

The database search did not reveal anything relevant to the attribute of *explanation*. To *explain* is to clarify the meaning of something to somebody (Encarta World English Dictionary, 2017), to make something clear in speech or writing, or to make plain by analysis or description (Wordsmyth English Dictionary).

Nothing explicit was found regarding the attribute *advice* in the database search; it was only used in an implicit way. *Advice* is remarks or an opinion offered as help in making a decision or in choosing a course of action (Wordsmyth English Dictionary), or a recommendation about action: somebody’s opinion about what another person should do (Encarta World English Dictionary, 2017).

#### Patient education

Both of the defining attributes that the COPD nurses mentioned of patient education, *teaching* and *learning*, were only used in an implicit way; nothing relevant to a concept analysis was found.

To *teach* is to instruct by imparting knowledge to someone (Wordsmyth English Dictionary) or to impart knowledge or skill to somebody by instruction or example (Encarta World English Dictionary, 2017).

To *learn* is to become informed of something (Wordsmyth English Dictionary), or to memorize something, such as a set of facts (Encarta World English Dictionary, 2017).

### Model case

A model case is an example of the use of a concept that demonstrates all the defining attributes of that concept (Walker & Avant, 2011). Here, the sub-concepts are used to demonstrate.

A woman with severe COPD was complaining to the COPD nurse that she couldn’t get to the store and do her shopping anymore. She became tired and experienced dyspnea. The dyspnea also frightened her. She lived in an apartment, two floors up. Because she could not do her shopping herself, she sent her husband or her grandchildren to the store, but she was not satisfied with this solution. She felt that there was always something missing in her larder or refrigerator. She wanted to go to the store herself and choose products from the shelves. The nurse educated the woman about her disease and tried to teach her a breathing technique known as ‘pursed-lip breathing’. The nurse sent her to the physiotherapist and the occupational therapist for further therapy, hoping that she could learn more about breathing techniques and techniques to conserve energy, and perhaps also learn that it would not be dangerous for her to perform physical activities. After a few months, the patient returned for a follow-up with the COPD nurse. Now she had found relief from her symptoms, and could do her shopping on her own. She did not do all her shopping at the same time, in order to make sure that her groceries were not too heavy to carry, and she used pursed-lip breathing when she climbed the stairs. She also took daily walks to improve her physical fitness. She was now happier and more satisfied with her life.

The nurse supervised the patient in pursed-lip breathing and had a revisit to check up on it when supervision is an ongoing process. The nurse gave her information about the physiotherapist and the occupational therapist, and educated her about her disease in a person-centred way; accordingly the nurse used Symptom-reducing actions.

### Additional cases

#### Borderline case

A borderline case is an example that contains most but not all of the defining attributes (Walker & Avant, 2011). The sub-concepts are also used here.

A 67-year-old woman underwent spirometry at the asthma/COPD clinic and was given a diagnosis of severe COPD. She had just moved to this town, and she told the nurse that she had attended an asthma/COPD clinic in her previous town. The nurse began to inform and educate the patient, as routine, about the disease, training, diet, and so on. The patient listened politely but before she left she said in a very low and quiet voice that she had heard this before and that she had been trying to live in the way that the nurse had described.

In this case, the COPD nurse provided the education and gave information about diet and training in a routine way, but did not check what stage the patient was at, and did not provide supervision. The nurse did not pay attention to the patient, and failed to adapt the education individually in a person-centred way.

#### Related case

Related cases are examples that are related to the concept but which do not contain the defining attributes, in this case the sub-concepts (Walker & Avant, 2011). Cases related to *supervision* might involve overseeing, those related to *information* might involve knowledge and instruction, and those related to *patient education* might involve pedagogy and training.

A 70-year-old man received a diagnosis of severe COPD and met with the nurse to be instructed in how to handle his inhaler. The nurse showed the man the inhaler and explained why he had to use it and when he could use it. She used a pedagogic approach, showing the man pictures and letting him ask questions if there was anything he did not understand. Then, the nurse instructed the man how to use the inhaler and let him try it while she oversaw the procedure. When the nurse could see that the man was able to handle the inhaler, and the man felt sure that he could do it, he went home and tried it on his own.

In this case the nurse took a pedagogic approach and gave the patient knowledge and instructions. She let him practise while she oversaw the procedure.

#### Contrary case

Contrary cases are examples of what the concept is not (Walker & Avant, 2011). Symptom-reducing actions are not health promotion or disease prevention.

The COPD nurse gave a lesson in a school to young people aged 13–15. He explained the dangers of smoking and the importance of avoiding smoking, and tried to get the students to reflect on the pros and cons. He spoke about some of the patients with COPD he had treated at the clinic, and described the problems they experienced. The nurse achieved good communication and dialogue with the young people, and many of them said at the end of the lesson that they would never start smoking because they did not want to have the problems associated with COPD.

This case is health promotion. The nurse’s activity was directed at healthy people in the expectation that they would avoid smoking and hence avoid COPD, and so would not need Symptom-reducing actions in the future.

### Antecedents

Antecedents are those events or incidents that must occur prior to the occurrence of the concept (Walker & Avant, 2011). *Unpleasant symptoms* are illness-related rather than treatment-related, and there may be relationships between them. Physiological, psychological, and situational factors affect the patient’s performance, including functional status, cognitive functions, and physical performance (Lenz, Suppe, Gift, Pugh, & Milligan, 1995). Impact of symptoms on function and health in patients with COPD are common (Theander et al., 2014). Symptoms can occur alone or in isolation from one another, but if multiple symptoms occur simultaneously they are likely to catalyse each other and further worsen the overall experience. Several dimensions are common across symptoms and clinical populations: intensity (strength and severity); timing (duration and frequency of occurrence); level of distress perceived (degree of discomfort or bothersomeness); and quality. People differ in their ability to discern symptoms (Lenz, Pugh, Milligan, Gift, & Suppe, 1997).

### Consequences

Consequences are those events or incidents that occur as a result of the occurrence of the concept; they are the outcomes of the concept (Walker & Avant, 2011). For Symptom-reducing actions, person-centred outcomes are important, such as control of symptoms, the ability to perform daily activities, exercise performance, and improved quality of life (ATS/ERS, 2006). Several COPD-specific instruments are available to measure the effectiveness of Symptom-reducing actions (ATS/ERS, 2006; Roman-Rodriguez, Pardo, Lopez, Ruiz, & Van Boven, 2016).

### Empirical referents

Empirical referents are classes or categories of actual phenomena that by their existence or presence demonstrate the occurrence of the concept itself (Walker & Avant, 2011). In this case, there are no empirical referents to the concept of Symptom-reducing actions. But there is a large amount of instruments to assess different symptoms in COPD such as health-related quality of life (van der Molen et al., 2003), dyspnea (Celli & MacNee, 2004), functional capacity (van Stel, Bogaard, Rijssenbeek-Nouwens, & Colland, 2001), fatigue (Theander, Jakobsson, Torstensson, & Unosson, 2008), and weight loss (WHO, 1995) for example.

## Discussion

### A summary of the concept analysis of Symptom-reducing actions

The three sub-concepts of supervision, information, and patient education, along with their defining attributes, constitute *Symptom-reducing actions*: a way to support the patient with COPD to perform self-care. The result of this analysis was that symptom-management is what the patients do and Symptom-reducing actions is what the nurses do when supporting the patients to manage their symptoms. An indication of illness felt by the patient could be decreased or simplified by the process of working toward a goal (Encarta World English Dictionary, 2017; Wordsmyth English Dictionary), specifically to manage self-care. The COPD nurse can achieve this via supervision, information, and patient education with an integrated emotional component. To perform symptom management is to transform unpleasant sensations to actions or non-actions (Henly et al., 2003). The COPD nurse promotes self-management and has strategies to reduce symptoms (Parker et al., 2005) by giving instructions and by motivating and supporting the patient with COPD in a dialogue where the patient’s own narrative is central, a person-centred approach (McCormack & McCance, 2006). COPD nurses give information in a communication process, and can perceive the ability to provide support, heighten awareness, and provide anticipatory guidance to those in their care (Erdley, 2005). The COPD nurse clarifies the meaning of something and recommends actions with advice. The nurse helps the patient to gain the motivation, skills, and confidence to control the disease COPD (Powell & Gibson, 2007) through a planned process of activities (Piredda, 2004), and plays the role of enabler (Stamler, 1996) in the process of patient education. They instruct by imparting knowledge to the patient with COPD, and help the patient to memorize facts and how to use the information. The process involves follow-ups on an individual basis.

### Interpretation of findings with implications for practice and future research

COPD is a disease that cannot be cured, but its symptoms can be reduced by providing patients with support regarding self-care and self-management (GOLD, 2015; Zwerink et al., 2014). It is perhaps often the case that nurses perform Symptom-reducing actions but are not aware of what they are doing; it is something they ‘just do’ in their daily work. The self-management support is often dependent on the nurses’ existing perceptions and knowledge (Lake & Staiger, 2010). This concept analysis can help them to be more aware of their task and the universities perhaps can offer more courses for nurses in pedagogics.

There was found two different nursing foci: individual-oriented and task-oriented (Lundh, Rosenhall, & Tornkvist, 2006). The individual-oriented nurses reflected more over their professional role and were more independent in their patient education. Perhaps a use of this concept analysis of Symptom-reducing actions can expand the nurses to reflect on their patient education.

It is not only the nurse who is responsible and important in patient education and Symptom-reducing actions. There is strong evidence that inter-professional collaboration in patient education to support self-management improves health-related quality of life and functional capacity, and decreases dyspnea (Kruis et al., 2013). It should be in any health professional’s interest to do Symptom-reducing actions out of their special competencies. Symptom-reducing actions are important for the patient with COPD to be supported from all health professionals.

A middle-range theory integrates theory and empirical research and can be a way to explain the observed regularities of social behaviour, organization, and social change. This concept analysis of *Symptom-reducing actions* may help nurses to reflect on their task of providing and organizing education for patients with COPD. It is valuable to know what nurses are doing, and why they are doing it, i.e., their social behaviour in education. This article could spark a discussion aimed at increasing the knowledge of Symptom-reducing actions and perhaps there is a need for a social change among nurses’ patient education.

Further investigation of what interpersonal and professional competence is could help develop the therapeutic use of self as a tool together with Symptom-reducing actions.

It is necessary to evaluate the outcomes of Symptom-reducing actions and its process by instruments for health-related quality of life (van der Molen et al., 2003), dyspnea (Celli & MacNee, 2004), functional capacity with 6-minutes walking-test (van Stel et al., 2001), fatigue with Fatigue Impact Scale (Theander et al., 2008), and weight loss by Body Mass Index (WHO, 1995) for example. According to Walker and Avant (2011), a concept analysis never can be seen as a finished product; the process must always continue.

### Strength and limitations of this study

The method of Walker and Avant (2011) gave a structured method to use in analysing the concept of Symptom-reducing actions. One difficulty was the number of articles that were found that only used the concepts in an implicit way. The limitation to ‘concept analysis’ could also have been a mistake which could mean that important information remained undetected. The concept of Symptom-reducing actions still lacks clarity. There is a need for more research into what it means for healthcare professionals and the nurses caring for patients with COPD in order to be a benefit for the patients.

## Conclusion

There is a difference between symptom-management and Symptom-reducing actions. Symptom-management is the patient’s challenge and Symptom-reducing actions are the nurse’s responsibility. Symptom-reducing actions in COPD are a way of mediating facts in a purposeful process performed by the nurse in order to enable the patient to take control over and manage unpleasant symptoms through communication and dialogue in participation, interaction, and partnership with an integrated emotional component. Time is needed for observation, identification, creating contact, and listening. The nurse has to use individual implementation and adaptation with flexibility and respect. This is a person-centred, planned process that enables and supports interpersonal competence, and requires the patient’s readiness and willingness. The process will increase the patient’s knowledge and skills in relation to his or her chronic illness.
